# Anaerobic RSH-dependent tellurite reduction contributes to *Escherichia coli tolerance* against tellurite

**DOI:** 10.1186/s40659-022-00383-5

**Published:** 2022-03-21

**Authors:** P. Muñoz-Diaz, K. Jiménez, R. Luraschi, F. Cornejo, M. Figueroa, C. Vera, A. Rivas-Pardo, J. M. Sandoval, C. Vásquez, F. Arenas

**Affiliations:** 1 Laboratorio Microbiología Molecular, Departamento de Biología, Facultad de Química Y Biología, Universidad de Santiago de Chile, Santiago, Chile; 2 Laboratorio de Genómica Microbiana, Centro de Genómica Y Bioinformática, Universidad Mayor, Santiago, Chile; 3 Facultad de Ciencias, Universidad Arturo Prat, Iquique, Chile

**Keywords:** Tellurite reduction, *Escherichia coli*, Glutathione, RSH

## Abstract

**Background:**

Tellurium is a rare metalloid that exerts high toxicity on cells, especially on bacteria, partly due to reactive oxygen species (ROS) generation. Moreover, it has also been observed that tellurite can target free cell thiols groups (RSH) (i.e. reduced glutathione (GSH)), enhancing the cellular redox imbalance. Additionally, in vitro experiments have suggested that several enzymes can reduce tellurite (IV) to its elemental form (0); where RSH present on their active sites may be responsible for the process. Nevertheless, the mechanisms implemented by bacteria for tellurite reduction and its role in resistance have not been evaluated in vivo.

**Results:**

This work shows that tellurite reduction to elemental tellurium is increased under anaerobic conditions in *E. coli* cells. The in vivo tellurite reduction is related to the intracellular concentration of total RSH, in the presence and absence of oxygen. This metabolization of tellurite directly contributes to the resistance of the bacteria to the oxyanion.

**Conclusions:**

We demonstrated that in vivo tellurite reduction is related to the intracellular thiol concentration, i.e. large availability of cellular RSH groups, results in a more significant reduction of tellurite. Furthermore, we observed that, when the bacterium exhibits less resistance to the oxyanion, a decreased tellurite reduction was seen, affecting the growth fitness. Together, these results let us propose that tellurite reduction and the intracellular RSH content are related to the oxyanion bacterial resistance, this tripartite mechanism in an oxygen-independent anaerobic process.

## Background

Tellurite, the most soluble and bioavailable tellurium oxyanion [13], exhibits high toxicity on *Escherichia coli,* exceeding the effect found on the minimum inhibitory concentration of other ions and toxic compounds such as AsO_2_^−^, AsO_4_^3−^, Hg^2+^, SiO_3_^2−^, Pb^2+^, SeO_3_^2−^, Cd^2+^, Cu^2+^, Ag^+^, and SeO_4_^2−^ [20, 52, 55]. Tellurite metabolization triggers protein oxidation (carbonylation) [17, 32], dismantling of [4Fe–4S] centers of dehydratases, such as AcnB and FumA [10], and membrane lipoperoxidation [32–34]. In addition, tellurite directly affects the synthesis of heme groups, producing the accumulation of the toxic intermediate protoporphyrin IX [28]. Moreover, other important cellular processes affected by tellurite include inhibition of glycolysis, tricarboxylic acid cycle, respiratory chain, and glutathione metabolism, among others [12, 19, 47–49].

Several molecules with RSH groups are also targets of tellurite, which are likely to participate directly in the oxyanion reduction [47, 48]. Exposure to high concentrations of cysteine or reduced glutathione (GSH) triggers the reduction of tellurite in vitro, evidenced by the formation of a black precipitate of Te^0^ in the assay [45, 46]. A possible explanation for this phenomenon is that tellurite and the thiol groups experience a Painter-like reaction (RSH + TeO_3_^2−^ → RS-Te-SR → RSSR + Te^0^) [31]. However, the intracellular concentration of free thiols molecules is very low [8], this reaction's progress must occur catalytically, possibly in collaboration with some of the tellurite reducing enzymes. In addition, cellular systems related to the metabolism of RSH molecules can also be targeted by tellurite. Such is the case of glutathione reductase and thioredoxin reductase and their reduced products glutaredoxins (A, B, and C, and thioredoxins (A and C, which are directly oxidized by the toxic oxyanion [11, 36, 39, 48].

In vitro tellurite reduction has been determined by using crude extracts of bacteria such as *Thermus thermophilus* HB8 [15], *Shewanella oneidensis* [22, 50], and *Aeromonas caviae* [12]. To date, numerous NAD(P)H-dependent proteins that reduce tellurite have been identified. Nitrate reductases (NarG and NarZ) from *E. coli*, *Paracoccus denitrificans, P. pantotrophus* and *R. sphaeroides* [6, 40], the E3 component of pyruvate dehydrogenase complex from *E. coli, Zymomonas mobilis, Streptococcus pneumoniae, Geobacillus stearothermophilus* V and *Aeromonas caviae* [12], NDH-II from *E. coli* [19] and of *Pseudomonas *spp. BNF22 glutathione reductase [37] are only a few examples of proteins that contribute to tellurite resistance. However, some doubts regarding tellurite reduction contribution to toxic resistance have emerged. At least in aerobic conditions, this tellurite reduction process produces ROS [10, 32], so it would not constitute an entirely favorable mechanism for the cell. In addition, it has been observed that both highly tellurite-resistant and tellurite-susceptible bacteria, can reduce it. Some bacteria able to reduce tellurite include phototrophs to heterotrophs, under both aerobic and anaerobic conditions, such as *Shewanella*, *Staphylococcus epidermidis*, *Rhodobacter sphaeroides*, *Rhodobacter capsulatus*, and *Halomonas* [1, 18, 25, 35, 44].

The complete absence of ROS, in anaerobiosis, is a condition where oxygen-sensitive cellular molecules are produced by the cell. An example of this is the FNR regulator which is rapidly oxidized in the presence of oxygen, losing its function [26]. In contrast, anaerobiosis provides a reducing environment that favors the metabolization of oxidized chemical species, such as tellurite. Under this condition, it can be postulated that the oxyanion would react favorably with RSH groups molecules if compared with aerobic conditions. This is because, in the presence of oxygen, the systems associated with thiol groups metabolism, such as glutathione reductase, thioredoxin reductase, glutaredoxins, and thioredoxins, are involved primarily in RSH redox homeostasis to restore their bioavailability [5, 21, 38]. Therefore, in absence of ROS production, RSH molecules could be interacting with tellurite and via Painter's reaction in which tellurite reduction would be favored.

In this work, we demonstrate that independent of molecular oxygen, a strong correlation between the intracellular RSH concentration availability with the bacterium tellurite reduction ability, which in turn is related to oxyanion cellular resistance.

## Results

**Fig. 1:**
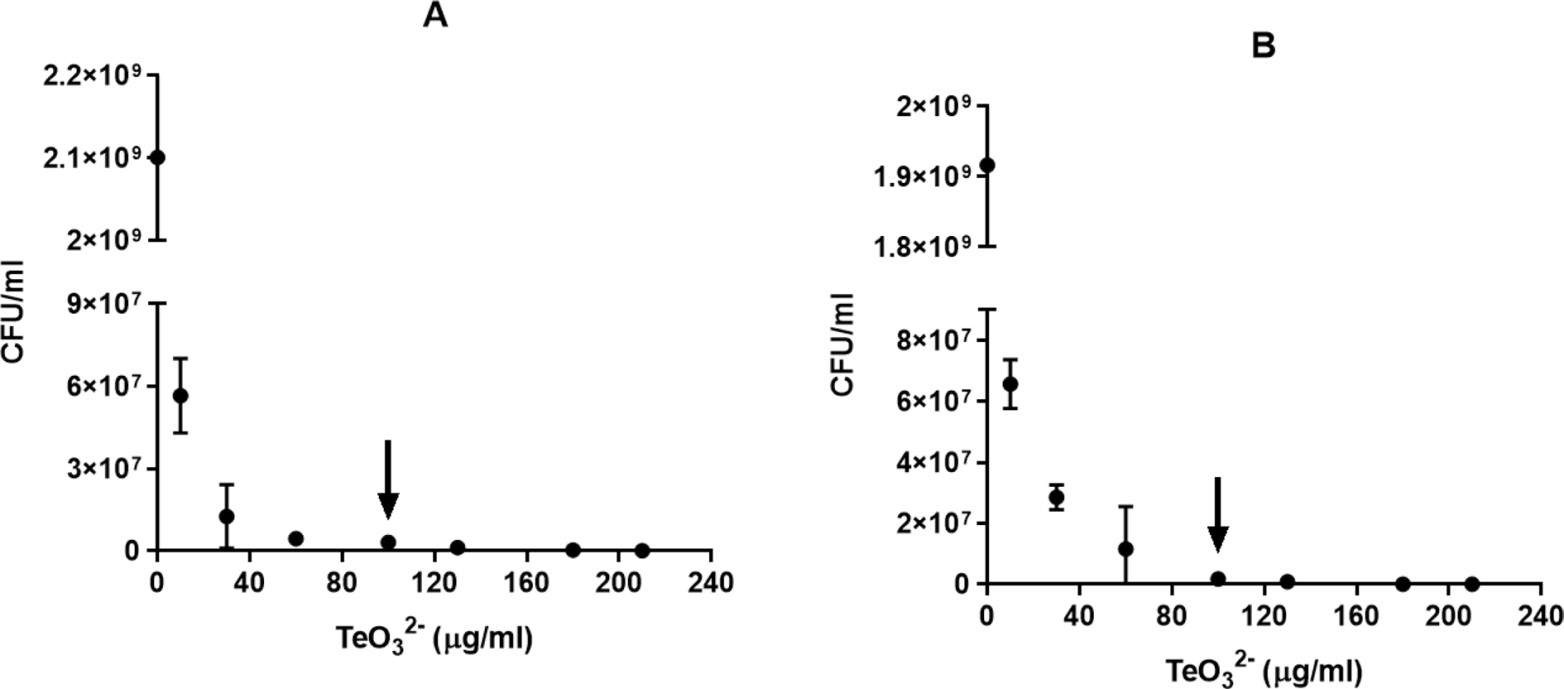
Determination of tellurite on cellular viability in anaerobic and aerobic growth conditions. The curves were obtained after 1 h of treatments with tellurite at different concentrations in anaerobiosis (**A**) or aerobiosis (**B**). The black arrows indicate the last concentration (100 µg/ml) at which viable cells were counted in both growth conditions. The results represent the average of 3 independent trials ± SD

Tellurite is an extremely toxic compound in bacteria, the minimum inhibitory concentration (MIC) of tellurite for *E. coli* in MOPS*n medium were 35 µg/ml and 110 µg/ml in aerobic and anaerobic conditions, respectively. To evaluate the effect of tellurite on the viability of *E. coli*, we conducted the treatments during the mid-exponential phase of the bacterial growth curve by calculating the Colony Forming Units *per* ml (CFU/ml; Fig. 1). At a tellurite concentration of 100 µg/ml, it was observed an equivalent effect in decreasing CFU/ml on both conditions (anaerobiosis and aerobiosis), so this concentration was chosen to analyze the following assays in vivo.

**Fig. 2:**
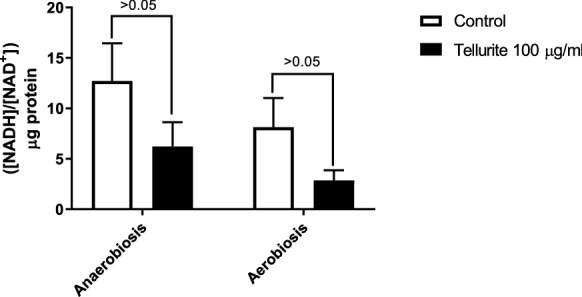
Effect of tellurite on NADH/NAD^+^ intracellular ratio in anaerobic and aerobic growth conditions. The ratio of reduced and oxidized nucleotide was normalized by protein concentration. The values on the bars correspond to the individual p-value of a two-way ANOVA analysis and post hoc analysis FDR correction. The results represent the average of 3 independent trials ± SD

As described, tellurite disturbs the redox homeostasis of the cell, so its oxidizing effect was analyzed on the intracellular NADH/NAD^+^ content ratio in both aerobic and anaerobic conditions of cell growth. Tellurite treatment decreases the NADH/NAD^+^ ratio significantly in an oxygen-independent fashion (Fig. 2). The quotient between both nucleotide species with or without tellurite is higher in anaerobic conditions, indicating that the baseline concentration of NADH is higher in the absence of oxygen and, in addition, that the result of the treatment in anaerobiosis has no significant difference from the baseline state of the quotient in aerobiosis. This result indicates that the redox imbalance is not oxygen dependent but is directly caused by the presence of tellurite.

**Fig. 3:**
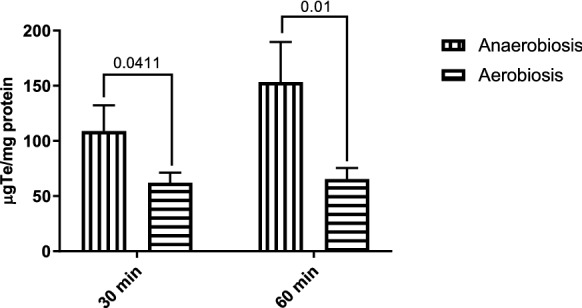
Determination of tellurium equivalents in bacterial sediments from *E. coli* growth in anaerobic and aerobic conditions. Bacteria were grown and treated with tellurite in the presence or absence of oxygen for 30 and 60 min and the intracellular content of tellurium was determined by ICP-OES atomic absorption spectrophotometry. The values on the bars correspond to the p-value obtained from one way-ANOVA and post hoc analysis FDR correction. The results represent the average of 3 independent trials ± SD

It is known that the tellurite exerts its toxicity inside the cell, however, it has not been evaluated whether the oxyanion uptake is affected by the presence or absence of oxygen. In Fig. 3, we show a higher uptake of tellurite at 30 and 60 min of treatment in anaerobic conditions. The higher resistance exhibited in this condition would not be related to this variable in terms of MIC. These results are in agreement with those obtained in the feasibility test, even there is greater oxyanion bioavailability under anaerobic conditions, where higher resistance to tellurite is exhibited.

**Fig. 4:**
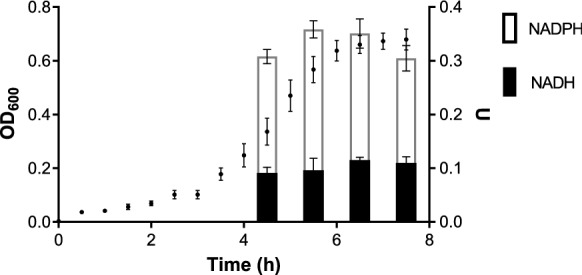
Nucleotide dependence for in vitro tellurite reduction in anaerobic growth curve. Tellurite reduction using protein crude extracts, samples at different points of growth in anaerobiosis were used in the presence of the indicated cofactors (NADPH or NADH). On the right Y-axis, U represents Tellurite reductase activity Units. The results represent the average of 3 independent trials ± SD

To determine at what moment of the bacterial growth curve occurs the highest tellurite reduction, samples at different optical densities under anaerobic conditions were tested. Crude protein extracts were prepared, and a tellurite reductase assay was assessed using 50 µg of total protein, NADH or NADPH. The results presented in Fig. 4 reveal a more significant reduction of tellurite in the presence of NADPH in the medium-late exponential phase (OD600 = 0.5). For this reason, it was decided to perform all the reduction tests in vivo in cultures in this growth phase.

**Fig. 5:**
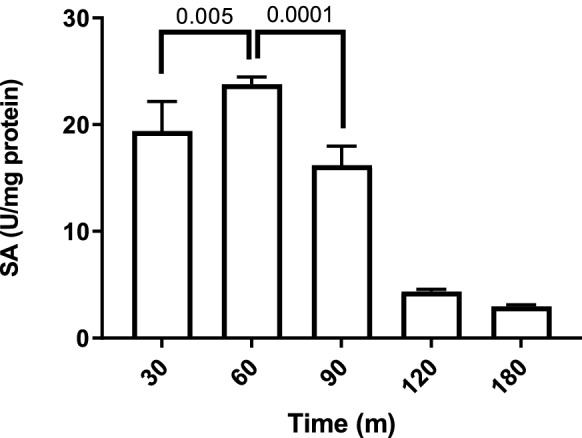
Anaerobic reduction of tellurite in vivo as a function of time. Bacterial cultures of *E. coli* BW25113 grown in anaerobiosis up to OD_600_ ~ 0.5 were used, which were treated with tellurite at a concentration of 100 µg/ml for the time indicated on the X-axis. The values on the bars correspond to the p-value of the statistical comparison between those that are contained in the marked interval. Statistical values were obtained by one-way ANOVA analysis and post hoc analysis FDR correction. The results represent the average of 3 independent trials ± SD

**Fig. 6:**
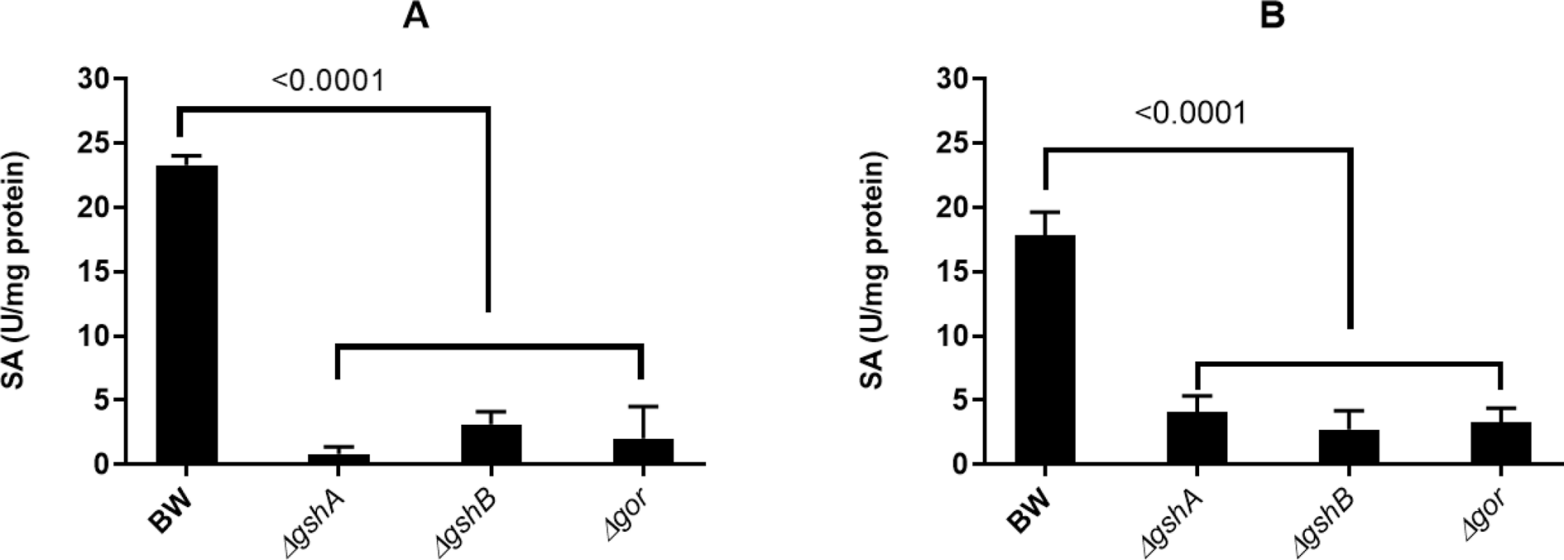
Specific activity of tellurite reductase in vivo in (**A**) anaerobiosis and (**B**) aerobiosis is decreased in GSH deficient strains. The strains tested are indicated next to the X-axis, tellurite reductase activity (U/mg protein) was determined. The p values < 0.05 indicate statistical significance corresponds to the ANOVA and post hoc analysis FDR correction. The results represent the average of 3 independent trials ± SD

**Fig. 7:**
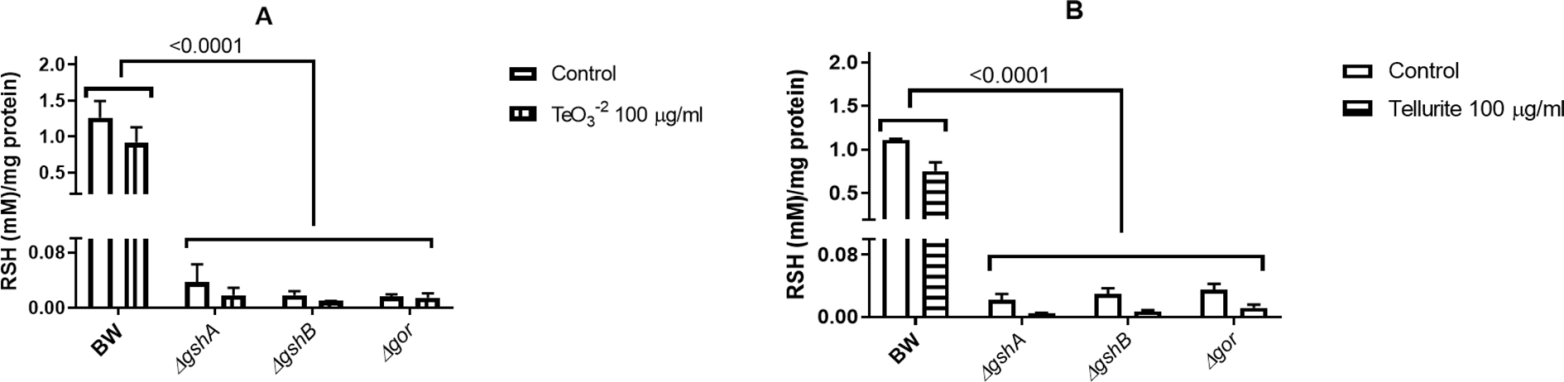
Content of RSH groups in anaerobiosis (**A**) and aerobiosis (**B**). The strains tested are indicated next to the X-axis, and total RSH concentration (mM/mg protein) was evaluated. The p values < 0.05 indicate statistical significance corresponds to the ANOVA and post hoc analysis FDR correction. The results represent the average of 3 independent trials ± SD

Tellurite reduction has not been quantitatively evaluated in vivo, mainly because the turbidity of the medium is affected by bacterial growth. As shown in Fig. 1, a change in optical density at high treatment concentrations is unlikely due to increased biomass; thus, the tellurite reduction at a 100 µg/ml concentration was evaluated. Figure 5 shows that the oxyanion reduction in anaerobiosis reaches a maximum at 60 min and then it declines progressively and remains constant at later times, so it was decided to test this time in the following assays. Next, crude extracts were obtained from three mutant strains involved in glutathione metabolism (Δ*gor*, Δ*gshA*, and Δ*gshB*) grown both in the presence or absence of oxygen, and the tellurite reduction was determined. As seen in Fig. 6, in both growth conditions, the highest reduction was exhibited by the wild-type strain BW25113, while the three mutants, all involved in glutathione metabolism, exhibit reduced in vivo reduction, implying that the tellurite reduction is directly related to the availability of reduced glutathione. To evaluate the bioavailability of total RSH groups in each of the mutants, we used the DTNB fluorescence method. Figure 7 summarized our findings, indicating a markedly lower availability of total RSH groups in the three mutant strains, independent of the growth condition, even without applying the tellurite treatment.

**Fig. 8:**
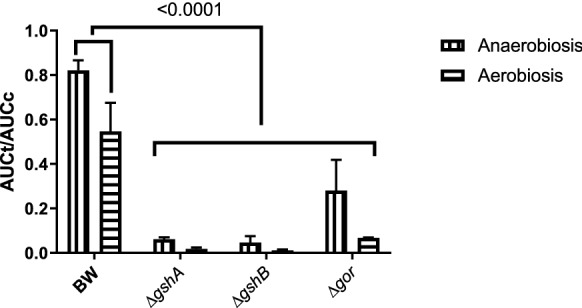
Area under the growth curve (AUC) of strains treated with tellurite in anaerobic and aerobic growth conditions. It was normalized by the area of the same strain without treatment. The value presented on the bars corresponds to the p-value obtained by the statistical comparison by ANOVA of a direction controlling FDR between the quotient of each strain and the ratio of BW25113 in the respective growth condition. The results represent the average of 3 independent trials ± SD

Finally, while the tellurite reduction is directly dependent on the concentration of total RSH groups and, apparently specific to GSH, a direct relationship between reduction and resistance to tellurite has not been established. Therefore, the effect on the growth curve of a sublethal tellurite concentration in both growth conditions was evaluated and the area under the curve (AUC) of each strain was obtained. As shown in Fig. 8, the three GSH deficient strains, which in turn have lower RSH content, exhibit lower cellular fitness than BW25113, indicating that this lower tellurite reduction capability is related to lower levels of RSH and which also implies that they are less resistant to the oxyanion.

## Discussion

The reduction of tellurite has been associated with its detoxification since no toxicity has been verified by the metalloid in its elemental state. The decrease of NADH and RSH was quite comparable in both conditions, the NADH/NAD^+^ ratio decreased by ~ 52% in anaerobiosis meanwhile, the decrease in aerobiosis was ~ 60%. Similarly, the RSH content showed a decrease of ~ 28%, and ~ 32% in anaerobiosis and aerobiosis, respectively (Figs. 2 and 7). Tellurite could promote redox events when reduced from tellurium (IV) to elemental tellurium (0), causing oxidation of NADH to NAD + and oxidation of RSH groups to RS-SR in this process. The observed decrease in NADH/NAD^+^ ratio and RSH content implies that tellurite still exerts an intracellular oxidative effect, already observed in aerobiosis, but not analyzed in anaerobiosis until this study.

As detectable molecular/biochemical features following the entry of tellurite in bacteria are oxidative macromolecular damage in proteins and/or lipid membrane, glutathione depletion, and decreased enzymatic activity; several of them are conditioned to be observed under aerobic growth conditions due to the generation of reactive oxygen species formation by the tellurite reduction process [32, 35, 47, 48]. A feature determined in a previous publication by our group was the effect of tellurite in the intracellular concentrations of NADH/NAD + and NADPH/NADP + on E. coli grown in aerobic conditions and we determined a reduction in the pool of NADH/NAD + by ~ 40% and an increased in the pool of NADPH/NADP + by ~ 25% [41]. In particular, the last effect only is triggered by the activation of the G6PDH enzyme in a ROS-dependent process mediated by soxRS regulon [41]. Under the anaerobic condition of growth tested in Fig. 4, we suspected that intracellular concentration of NADPH/NADP + could not be induced due to the absence of ROS formation triggered by tellurite reduction by several protein activities involved in the process. Additionally, NADPH concentration under anaerobic conditions of growth is considered to be maintained by the malic enzyme (part of the anaplerotic node: PEP-pyruvate-oxalacetate node), however, its exact contribution either in total NADPH pool, physiological regulation, and carbon flux is still needed to be completely established [43].

NADPH is one of the most important antioxidant cellular factors and participates in this process through several pathways [53], one of them is the regeneration of GSH from GSSG by glutathione reductase (Gor) enzyme [42, 53]. GSH plays a fundamental role in cellular redox homeostasis, by functioning as an electron donor reducing disulfide bonds in proteins [42, 56], fighting oxidative stress associated with metals such as mercury, cadmium silver, and selenium [3] or by chlorine [14]. Treatment with tellurite produces a significant decrease in GSH concentration, directly relating this tripeptide to tellurite with the formation of GS-Te-SG (tellurodiglutathione) intermediate, which would be a substrate for oxidoreductases, producing Te^0^ and GSSG as products [47], however, this has not been yet verified. The formation of this intermediate would explain the need for GSH to be present and its availability within the cell, which would affect tellurite reduction. All the mutant strains used in this study, showed a dramatic decrease (> 95% in the total RSH concentration when compared with the parental wild type strain; for Δ*gshA* and Δ*gshB* mutant strains lack the two genes encoding glutathione biosynthetic enzymes so their absence implies no availability of this molecule. Meanwhile, the Δ*gor* strain lacks the enzyme responsible for reconstituting GSH from its oxidized form (GSSG), which implies that the smallest tellurite reduction is not due to the absence of total glutathione, but specifically to its reduced form, GSH [42]. It is possible to assume that the lack of detectable RSH in these strains, GSH represents most of the RSH molecules detected by the technique utilized. The large depletion of GSH in mutant strains seems to immediately decrease the entire complement of RSH groups at the cellular level. Nearly 99.5% of glutathione exists as GSH, meanwhile, GSSG represents <  < 1%, with other forms of mixed disulfides and low weight RSH-compounds, adding up to the complete cellular thiol concentration in *E. coli* cells [42]. So, either the absence or decrease of GSH reduces the ability of cells to perform the tellurite reduction process in vivo (Figs. 6 and 7).

Tellurite reduction is one of several processes been identified as a mechanism that decreases the toxic effects of tellurite at the cellular level. However, we and others had shown that under aerobic conditions, where the assays are usually determined, different ROS are formed as by-products. As a result, it is still a controversial topic to determine whether for this oxyanion the process of its reduction is a molecular/physiological mechanism of resistance [13, 44]. When comparing the effect of tellurite in aerobic and anaerobic conditions, a similar decrease in cell viability was determined (Fig. 1). This result is associated with a significant reduction of both NADH/NAD^+^ ratio and total RSH concentrations, and as a phenotypical consequence (Figs. 2 and 7). Especially in the aerobic assays, these effects are more dramatic when compared with the anaerobic assays, which seem to be not related to the ability to incorporate intracellular tellurium (Fig. 3). As a possible explanation for this phenomenon could be mentioned that the reduction of oxyanion can be quickly transformed to a less toxic form by decreasing either its mobility and/or bioavailability. These mechanisms have been previously reported for selenium oxyanions (selenite and selenite), where bacteria reduce them to Se^0^ or methylated compounds via non-enzymatic and enzymatic processes [16, 29]. In some bacterial species, selenite oxyanions are used as a final electron acceptor by specific selenite reductases in anaerobic conditions [29, 30].

In vitro reduction of tellurite is a process carried out using purified proteins or crude protein extracts [4, 6, 12, 15, 19, 22, 37, 40, 50]. Limited studies have determined the role of low molecular mass such as GSH, and other RSH molecules (cysteine, *N*-acetyl cysteine, Coenzyme-A reduced form) had been identified as direct targets by the oxyanion [47], for this reason mostly all the responsibility in the reductase process has been attributed to enzymes [4]. In this work, the cell cultures were treated as a whole reductive environment, which is affected by the absence of any protein that may or may not modify, to some degree, the tellurite reduction mechanism (Figs. 6 and 7). Recently, it has been proposed a relationship between the cell reduction potential and the tellurite resistance in *Deinococcus radiodurans* [2].

Any genetic modification in a particular strain produces metabolic/physiological/chemical consequences at the cellular level that can affect various functions, but in this case, is focused on direct combat against a toxic substance in lethal concentrations. In particular, the strains that had a decreased GSH content were highly affected by tellurite according to the AUC assay (Fig. 8), due to reduced cell fitness [23]. Taken together, the results presented directly relate intracellular GSH to the ability to reduce oxyanion to a less toxic form and, as a consequence, its contribution to tellurite resistance.

## Conclusion

Under anaerobic, oxygen-independent growth conditions, the tellurite reductase activity contributes significantly to higher cellular fitness, this activity is enabled by the intracellular redox content, especially by RSH groups and glutathione GSH/total thiol concentration. In this work, independent of molecular oxygen a tripartite relationship between metalloid reduction, its resistance, and glutathione is established.

## Methods

### Strains and culture media

For all in vivo assays, MOPS buffered culture medium supplemented with 0.2% glucose (w/v) and 40 mM potassium nitrate (MOPS*n) was used. The in vitro assays were carried out with cell extracts obtained from cultures grown in LB medium supplemented with chloramphenicol. *Escherichia coli* BW25113 (*E. coli* WT, parental) was used as a wild strain. Additionally, the strains *Δgor, ΔgshA,* and *ΔgshB* from the Keio collection (NARA Institute, Japan) [7] were used in the in vivo reduction assay, RSH quantification, fitness determination, and ex vivo assay.

### Growth conditions

The cell cultures were grown in the presence or absence of oxygen. For the condition of anaerobiosis, an anaerobiosis chamber was used keeping an atmosphere of 100% N_2_ (Coy chamber). All cultures were grown at 37 °C with agitation. To ensure the absence of oxygen during bacterial growth, all cultures were started using a pre-inoculum that was prepared from a plate culture in an aerobic medium and incubated for at least 16 h in the absence of oxygen. Likewise, the culture medium in which the pre-inoculum was seeded was purged of oxygen using the pre-chamber of the Coy chamber with the semi-open bottles and kept inside the chamber for 12 h before use.

### Growth curves

Saturated bacterial cultures incubated overnight at 37 °C with agitation in aerobiosis or anaerobiosis, as appropriate, were used as pre-inoculum. Plates were used in whose wells was added 0 or 10 µg/ml of tellurite, 10 µL of saturated bacterial culture and completed at 1 mL volume with fresh MOPS*n medium. The curve was performed on the plate reader Tecan Infinite M200 Pro, at 37 °C with stirring at 150 rpm and measurements at 600 nm every 15 min for a total of 40 cycles. The curves were analyzed via R, obtaining the value of the area under the curve of each growth curve.

### Quantification of the uptake of tellurite into the cell

The proportion of total tellurium present in the cells (sediment), in the medium (supernatant), and adsorbed on the membrane (washed) was determined by ICP-OES atomic absorption spectrophotometry. The cells were cultured in the presence or absence of oxygen to OD_600_ ~ 0.5 and then treated with tellurite 50 or 100 µg/ml for 30 or 60 min. After treatment, 1 mL of culture was taken for protein quantification and 5 mL for the assay. The 5 mL was centrifuged at 9000×g for 5 min recovering the supernatant. The sediment was immediately washed with 5 mL of 50 mM Tris–HCl pH 7.4 and centrifuged again recovering the 5 mL of wash. A volume of 65% HNO_3_ was added to both 5 mL of supernatant and wash so that the final acid concentration was 2%. The bacterial sediment was also treated with 65% HNO_3_ (final concentration, 2%) and incubated at 37 °C for 2 h with stirring. Then, the dissolved sediment was flushed to 5 mL with 50 mM Tris–HCl pH 7.4, so that the final acid concentration was 2%. Sediment, supernatant, and wash were filtered using 0.22 µm Nylon filters and stored at 4 °C until analysis. The standards were prepared using tellurite dissolved in 2% nitric acid; the standards used were 0.01; 0.1; 0.5; 1.0; 10.0; 100.0 and 250 µg/ml. The results obtained were normalized by protein concentration.

### Determination of the viability percentage

The percentage of bacterial viability versus treatment with tellurite was performed in cultures by the micro drop method. Cultures of *E. coli* OD_600_ ~ 0.5 obtained as described above were plated and treated with increasing concentrations of tellurite in the range between 0 and 250 µg/ml. After the toxic addition, the cultures were incubated at 37 °C for 30 or 60 min. Of these, a 20 µL aliquot was taken that was diluted in 180 µL saline (0.9% NaCl w/v). From that dilution, serial dilutions were made 1/10 with saline solution until dilution 10^–6^. Then 4 µL of each dilution and the original culture were taken and deposited in drop form on LB agar plates 2% w/v. The plates were incubated at 37 °C for 16 h; subsequently, colonies were counted in the lowest dilution that contained countable colonies. The viability percentage was defined in relation to dilutions left untreated.

### NAD^+^/NADH ratio determination

The quantification was performed by the MTT method as described previously [24]. Briefly, sediments obtained from 10 ml of treated or untreated cultures in the presence or absence of oxygen were suspended in 2 mL of PBS. The volume was separated into 2 tubes that were centrifuged at 14,000×*g* for 5 min, discarding the supernatant. The sediment was suspended in 125 µL of 0.2 M HCl (NAD^+^ extraction) or 0.2 M NaOH of (NADH extraction). Then, the suspensions were heated at 100 °C for 10 min and immediately centrifuged as before. A calibration curve was prepared using commercial NADH or NAD^+^ of concentration 1.5 to 0.02 mM. With the samples and calibration curve prepared, the following solutions were prepared, which were applied in each reaction well in the indicated volumes; (1) Mix1, containing 20 µL of 1 M Bicin pH 8.0; 50 µL neutralization buffer (0.1 M HCl for NADH, 0.1 M NaOH for NAD^+^); 20 µL of 40 mM EDTA pH 8.0; 20 µL of 100% ethanol; (2) Mix2, containing 20 µL of PES (phenazine ethosulfate) 16 mM; 20 µL MTT (3-[4,5-dimethylthiazol-2-y] 2,5-diphenyltetrazolium bromide). From the tube for extraction of NAD^+^ 50 µL was taken and mixed with 4 µL of ADHII (yeast alcohol dehydrogenase II). The plate was incubated in the dark at 30 °C with stirring for 25 m. Once the incubation period elapsed, the reaction wells containing 50 µL of extract (NADH or NAD^+^), 110 µL of Mix1, and 40 µL of Mix2 were added. The absorbance measurement was started immediately at 570 nm every 30 s with stirring intervals using the Tecan Infinite M200 PRO reader. The calibration curve was constructed and the concentration in the samples was determined. The NADH and NAD^+^ concentrations obtained were normalized by protein concentration.

### Quantification of total reduced thiols (RSH).

*E. coli* cultures were incubated to OD_600_ ~ 0.5 and separated in identical volume samples, one was treated with tellurite 100 µg/mL and one was left untreated (control); both groups were incubated for 1 h at 37 °C with shaking at 150 rpm in the initial growth condition. At the end of the treatment, 500 µL aliquots were taken and centrifuged at 14,000×*g* for 1 min, discarding the supernatant. The sediments were stored at − 20 °C until the time of measurement. Each sample was suspended in 1 mL of RSH buffer, containing 50 mM Tris–HCl pH 8.0, 5 mM EDTA, 0.1% SDS and 0.1 mM DTNB (5,5'-dithiobis (2-nitrobenzoic acid)). The suspension was incubated at 37 °C for 30 min, homogenized in a vortex for 10 s and centrifuged for 10 min at 14,000×*g*. Subsequently, the absorbance of the supernatant of each fraction obtained was evaluated spectrophotometrically at 412 nm. Using the extinction coefficient molar of oxidized DTNB (1.36 × 10^4^ M^−1^ cm^−1^), the concentration of reduced thiols was determined. The determined RSH concentrations were normalized by protein concentration.

### Protein quantification

For this purpose, the Bradford method was used [9], repeating the calibration curve protocol, that is, in 150 µL wells 150 µL of Bradford reagent, 1–10 µL of protein sample and water miliQ completing the volume of the well. The determination was made on the Tecan Infinite M200 Pro plate reader by recording the absorbance of the sample at 595 nm.

### Determination of specific tellurite reducing activity in vitro

This was determined using crude protein extracts and/or purified proteins. In both cases tellurite reductase buffer was used [12, 37], changing the pH of the reaction (20 mM sodium phosphate buffer (pH 7.0 and 8.0) or Tris–HCl buffer (pH 9.0), 0.1 mM NADH and/or NADPH, 1 mM β-mercaptoethanol and 0.5 µg/mL potassium tellurite) and 40 µg of protein. The reaction was performed on the Tecan Infinite M200 pro reader, evaluating the absorbance at 500 nm at 37 °C for 15 min for purified protein or 60 min for crude extracts. An enzyme unit (U) was defined as the amount of enzyme needed to increase the absorbance at 500 nm in 0.001 units in 1 min under the test conditions; the specific activity (SA) was determined normalized by the protein concentration (U/mg protein).

### Determination of tellurite reduction in vivo

Cultures initiated from saturated pre-inoculums prepared at least 16 h in advance, were incubated in the measurement condition (aerobiosis or anaerobiosis, 37 °C, and agitation at 150 rpm) up to OD_600_ ~ 0.5 (the acceptable range it was considered between 0.45 and 0.55). At that point, the cultures were treated with 100 µg/ml tellurite, and the initial absorbance at 600 nm and 500 nm was immediately evaluated. The cultures were incubated at 37 °C for 1 h with shaking at 150 rpm, at the end of this period the absorbance at 500 nm was evaluated again. An enzyme unit (U) was defined as the amount of enzyme needed to increase absorbance at 500 nm in 0.001 units in 1 min under the test conditions; the specific activity (SA) was determined by dividing U by the concentration of proteins (U/mg/ml proteins).

### Data analysis

Statistical analysis and graphs were carried out using GraphPad Prism 6.0 (GraphPad Software, Inc.). The confidence interval in the analysis of variance (ANOVA) was set at p < 0.05 with post hoc analysis false discovery rate (FDR) correction.

## Data Availability

All data generated or analyzed during this study are included in this published article.

## Abbreviations

ROS: Reactive oxygen species
MIC: Minimal inhibitory concentration
GSH: Glutathione
AUC: Area under the curve
RSH: Thiol groups
CFU/ml: Colony Forming Units per ml
WT: Wild type strain
GSSG: Oxidized glutathione
FDR: False discovery rate

## References

[1] Amoozegar MA, Ashengroph M, Malekzadeh F (2008). Isolation and initial characterization of the tellurite reducing moderately halophilic bacterium, *Salinicoccus* sp. strain QW6. Microbiol Res.

[2] Anaganti N, Basu B, Gupta A (2015). Depletion of reduction potential and key energy generation metabolic enzymes underlies tellurite toxicity in *Deinococcus radiodurans*. Proteomics.

[3] Apontoweil P, Berends W (1975). Glutathione biosynthesis in *Escherichia coli* K 12 properties of the enzymes and regulation. Biochem Biophys Acta.

[4] Arenas-Salinas M, Vargas-Pérez JI, Morales W (2016). Flavoprotein-mediated tellurite reduction: structural basis and applications to the synthesis of tellurium-containing nanostructures. Front Microbiol.

[5] Åslund F, Ehn B, Miranda-Vizuete A (1994). Two additional glutaredoxins exist in *Escherichia coli*: Glutaredoxin 3 is a hydrogen donor for ribonucleotide reductase in a thioredoxin/glutaredoxin 1 double mutant. Proc Natl Acad Sci USA.

[6] Avazeri C, Turner RJ, Pommier J (1997). Tellurite reductase activity of nitrate reductase is responsible for the basal resistance of *Escherichia coli* to tellurite. Microbiology.

[7] Baba T, Ara T, Hasegawa M (2006). Construction of *Escherichia coli* K-12 in-frame, single-gene knockout mutants: the Keio collection. Mol Syst Biol.

[8] Bennett BD, Kimball EH, Gao M (2009). Absolute metabolite concentrations and implied enzyme active site occupancy in *Escherichia coli*. Nat Chem Biol.

[9] Bradford MM (1976). A rapid and sensitive method for the quantitation of microgram quantities of protein utilizing the principle of protein-dye binding. Anal Biochem.

[10] Calderón IL, Arenas FA, Pérez JM (2006). Catalases are NAD(P)H-dependent tellurite reductases. PLoS ONE.

[11] Carmel-Harel O, Storz G (2000). Roles of the glutathione- and thioredoxin-dependent reduction systems in the *Escherichia coli* and *Saccharomyces cerevisiae* responses to oxidative stress. Annu Rev Microbiol.

[12] Castro ME, Molina R, Díaz W (2008). The dihydrolipoamide dehydrogenase of *Aeromonas caviae* ST exhibits NADH-dependent tellurite reductase activity. Biochem Biophys Res Commun.

[13] Chasteen TG, Fuentes DE, Tantaleán JC (2009). Tellurite: History, oxidative stress, and molecular mechanisms of resistance. FEMS Microbiol Rev.

[14] Chesney JA, Eaton JW, Mahoney JR (1996). Bacterial glutathione: a sacrificial defense against chlorine compounds. J Bacteriol.

[15] Chiong M, Barra R, González E, Vásquez C (1988). Resistance of *Thermus* spp. to potassium tellurite. Appl Environ Microbiol.

[16] Combs GF, Jr Garbisu C, Yee BC (1996). Bioavailability of selenium accumulated by selenite-reducing bacteria. Biol Trace Elem Res.

[17] Contreras N, Vásquez CC (2010). Tellurite-induced carbonylation of the *Escherichia coli* pyruvate dehydrogenase multienzyme complex. Arch Microbiol.

[18] Cooper PD, Few AV (1952). Uptake of potassium tellurite by a sensitive strain of *Escherichia coli*. Biochem J.

[19] Díaz-Vásquez WA, Abarca-Lagunas MJ, Cornejo FA (2015). Tellurite-mediated damage to the *Escherichia coli* NDH-dehydrogenases and terminal oxidases in aerobic conditions. Arch Biochem Biophys.

[20] Harrison JJ, Ceri H, Stremick CA (2004). Biofilm susceptibility to metal toxicity. Environ Microbiol.

[21] Imlay James A (2003). Pathways of oxidative damage. Annu Rev Microbiol.

[22] Klonowska A, Heulin T, Vermeglio A (2005). Selenite and tellurite reduction by *Shewanella oneidensis*. Appl Environ Microbiol.

[23] Koseki S, Nonaka J (2012). Alternative approach to modeling bacterial lag time, using logistic regression as a function of time, temperature, pH, and sodium chloride concentration. Appl Environ Microbiol.

[24] Leonardo MR, Dailly Y, Clark DP (1996). Role of NAD^+^ in regulating the *adhE* gene of *Escherichia coli*. J Bacteriol.

[25] Maltman C, Yurkov V (2019). Extreme environments and high-level bacterial tellurite resistance. Microorganisms.

[26] Moore LJ, Kiley PJ (2001). Characterization of the dimerization domain in the FNR transcription factor. J Biol Chem.

[27] Moore MD, Kaplan S (1992). Identification of intrinsic high-level resistance to rare-earth oxides and oxyanions in members of the class Proteobacteria: characterization of tellurite, selenite, and rhodium sesquioxide reduction in *Rhodobacter sphaeroides*. J Bacteriol.

[28] Morales EH, Pinto CA, Luraschi R (2017). Accumulation of heme biosynthetic intermediates contributes to the antibacterial action of the metalloid tellurite. Nat Commun.

[29] Nancharaiah YV, Lens PN (2015). Ecology and biotechnology of selenium-respiring bacteria. Microbiol Mol Biol Rev.

[30] Narasingarao P, Häggblom MM (2007). Identification of anaerobic selenate-respiring bacteria from aquatic sediments. Appl Environ Microbiol.

[31] Painter EP (1941). The chemistry and toxicity of selenium compounds, with special reference to the selenium problem. Chem Rev.

[32] Pérez JM, Calderón IL, Arenas FA (2007). Bacterial toxicity of potassium tellurite: unveiling an ancient enigma. PLoS ONE.

[33] Pradenas GA, Díaz-Vásquez WA, Pérez-Donoso JM (2013). Monounsaturated fatty acids are substrates for aldehyde generation in tellurite-exposed *Escherichia coli*. BioMed Res Intern.

[34] Pradenas GA, Paillavil BA, Reyes-Cerpa S (2012). Reduction of the monounsaturated fatty acid content of *Escherichia coli* results in increased resistance to oxidative damage. Microbiology (Reading, England).

[35] Presentato A, Turner RJ, Vásquez CC (2019). Tellurite-dependent blackening of bacteria emerges from the dark ages. Environ Chem.

[36] Prinz WA, Åslund F, Holmgren A (1997). The role of the thioredoxin and glutaredoxin pathways in reducing protein disulfide bonds in the *Escherichia coli* cytoplasm. J Biol Chem.

[37] Pugin B, Cornejo FA, Muñoz-Díaz P (2014). Glutathione reductase-mediated synthesis of tellurium-containing nanostructures exhibiting antibacterial properties. Appl Environ Microbiol.

[38] Reeves SA, Parsonage D, Nelson KJ (2011). Kinetic and thermodynamic features reveal that *Escherichia coli* BCP is an unusually versatile peroxiredoxin. Biochemistry.

[39] Rigobello MP, Folda A, Citta A (2011). Interaction of selenite and tellurite with thiol-dependent redox enzymes: kinetics and mitochondrial implications. Free Radic Biol Med.

[40] Sabaty M, Avazeri C, Pignol D (2001). Characterization of the reduction of selenate and tellurite by nitrate reductases. Appl Environ Microbiol.

[41] Sandoval JM, Arenas FA, Vásquez CC (2011). Glucose-6-phosphate dehydrogenase protects *Escherichia coli* from tellurite-mediated oxidative stress. PLoS ONE.

[42] Smirnova GV, Oktyabrsky ON (2005). Glutathione in bacteria. Biochemistry (Mosc).

[43] Spaans SK, Weusthuis RA, van der Oost J (2015). NADPH-generating systems in bacteria and archaea. Front Microbiol.

[44] Summers AO, Jacoby GA (1977). Plasmid-determined resistance to tellurium compounds. J Bacteriol.

[45] Taylor DE (1999). Bacterial tellurite resistance. Trends Microbiol.

[46] Tucker FL, Thomas JW, Appleman MD (1962). Complete reduction of tellurite to pure tellurium metal by microorganisms. J Bacteriol.

[47] Turner RJ, Aharonowitz Y, Weiner JH (2001). Glutathione is a target in tellurite toxicity and is protected by tellurite resistance determinants in *Escherichia coli*. Can J Microbiol.

[48] Turner RJ, Weiner JH, Taylor DE (1999). Tellurite-mediated thiol oxidation in *Escherichia coli*. Microbiology.

[49] Valdivia-González M, Pérez-Donoso JM, Vásquez CC (2012). Effect of tellurite-mediated oxidative stress on the *Escherichia coli* glycolytic pathway. Biometals.

[50] Valdivia-González MA, Díaz-Vásquez WA, Ruiz-León D (2018). A comparative analysis of tellurite detoxification by members of the genus *Shewanella*. Arch Microbiol.

[51] Vásquez CC, Saavedra CP, Loyola CA (2001). The product of the *cysK* gene of *Bacillus stearothermophilus* V mediates potassium tellurite resistance in *Escherichia coli*. Curr Microbiol.

[52] Workentine ML, Harrison JJ, Stenroos PU (2008). *Pseudomonas fluorescens*' view of the periodic table. Environ Microbiol.

[53] Ying W (2008). NAD^+^/NADH and NADP^+^/NADPH in cellular functions and cell death: regulation and biological consequences. Antioxid Redox Signal.

[54] Yurkov V, Jappe J, Vermeglio A (1996). Tellurite resistance and reduction by obligately aerobic photosynthetic bacteria. Appl Environ Microbiol.

[55] Zannoni D, Borsetti F, Harrison JJ (2008). The bacterial response to the chalcogen metalloids Se and Te. Adv Microb Physiol.

[56] Zhao Y, Seefeldt T, Chen W (2009). Effects of glutathione reductase inhibition on cellular thiol redox state and related systems. Arch Biochem Biophys.

